# MassTag Polymerase Chain Reaction for Differential Diagnosis of Viral Hemorrhagic Fevers

**DOI:** 10.3201/eid1204.051515

**Published:** 2006-04

**Authors:** Gustavo Palacios, Thomas Briese, Vishal Kapoor, Omar Jabado, Zhiqiang Liu, Marietjie Venter, Junhui Zhai, Neil Renwick, Allen Grolla, Thomas W. Geisbert, Christian Drosten, Jonathan Towner, Jingyue Ju, Janusz Paweska, Stuart T. Nichol, Robert Swanepoel, Heinz Feldmann, Peter B. Jahrling, W. Ian Lipkin

**Affiliations:** * Columbia University, New York, New York, USA;; † University of Pretoria and National Health Laboratory Services, Pretoria, South Africa;; ‡ Public Health Agency of Canada, Winnipeg, Manitoba, Canada;; § United States Army Medical Research Institute of Infectious Diseases, Fort Detrick, Frederick, Maryland, USA;; ¶ Bernhard-Nocht-Institute of Tropical Medicine, Hamburg, Germany;; # Centers for Disease Control and Prevention, Atlanta, Georgia, USA;; ** National Institute for Communicable Diseases, Sandringham, South Africa;; †† University of Manitoba, Winnipeg, Manitoba, Canada;; ‡‡ National Institutes of Allergy and Infectious Diseases Integrated Research Facility, Fort Detrick, Frederick, Maryland, USA

**Keywords:** Mass spectroscopy, PCR, multiplex, viral hemorrhagic fever, differential diagnosis, infectious diseases

## Abstract

Viral hemorrhagic fevers are associated with high rates of illness and death. Although therapeutic options are limited, early differential diagnosis has implications for containment and may aid in clinical management. We describe a diagnostic system for rapid, multiplex polymerase chain reaction identification of 10 different causes of viral hemorrhagic fevers.

Increasing international travel, trafficking in wildlife, political instability, and terrorism have made emerging infectious diseases a global concern. Viral hemorrhagic fevers (VHF) warrant specific emphasis because of their high rates of illness and death, and the potential for rapid dissemination by human-to-human transmission. The term "viral hemorrhagic fever" characterizes a severe multisystem syndrome associated with fever, shock, and bleeding diathesis caused by infection with any of several RNA viruses, including Ebola virus and Marburg virus (MARV) (family *Filoviridae*); Lassa virus (LASV) and the South American hemorrhagic fever viruses Guanarito virus, Junín virus, Machupo virus, and Sabiá virus (*Arenaviridae*); Rift Valley fever virus (RVFV), Crimean-Congo hemorrhagic fever virus (CCHFV), and hantaviruses (*Bunyaviridae*); and Kyasanur Forest disease virus (KFDV), Omsk hemorrhagic fever virus, yellow fever virus (YFV), and dengue viruses (*Flaviviridae*) (*1**,**2*). Although clinical management of VHF is primarily supportive, early diagnosis is needed to contain the contagion and implement public health measures, especially if agents are encountered out of their natural geographic context.

Vaccines have been developed for YFV, RVFV, Junín virus, KFDV, and hantaviruses (*3**–**7*), but only YFV vaccine is widely available. Early treatment with immune plasma was effective in Junín virus infection (*8*). The nucleoside analog ribavirin may be helpful if given early in the course of Lassa fever (*9*), Crimean-Congo hemorrhagic fever (*10*), or hemorrhagic fever with renal syndrome (*11*) and is recommended in postexposure prophylaxis and early treatment of arenavirus and bunyavirus infections (*12*).

Methods for direct detection of nucleic acids of microbial pathogens in clinical specimens are rapid, sensitive, and obviate the need for high-level biocontainment. Numerous systems are described for nucleic acid detection of VHF agents; however, none are multiplex (*13*). Although geographic location or travel history of suspected patients usually restricts the number of agents to be considered, diagnosis of VHF may be difficult in case of an intentional release (*12*). Symptoms of VHF are initially nonspecific and may include fever, headache, myalgia, and gastrointestinal or upper respiratory tract complaints (*1*); thus, assays that allow simultaneous consideration of multiple agents are needed.

We recently described the application of MassTag polymerase chain reaction (PCR) in the context of differential diagnosis of respiratory disease (*14*). MassTag PCR is a multiplex assay in which microbial gene targets are coded by a library of 64 distinct mass tags. Nucleic acids (RNA or DNA) are amplified by multiplex (reverse transcription–) PCR using up to 64 primers, each labeled by a photo-cleavable link with a different molecular weight tag. After separation of the amplification products from unincorporated primers and release of the mass tags from the amplicons by UV irradiation, tag identity is analyzed by mass spectrometry. The identity of the microbe in the clinical sample is determined by the presence of its 2 cognate tags, 1 from each primer.

## The Study

To facilitate rapid differential diagnosis of VHF agents, we established the Greene MassTag Panel VHF version 1.0, which comprises the following targets: Ebola Zaire virus (ZEBOV), Ebola Sudan virus (SEBOV), MARV, LASV, RVFV, CCHFV, Hantaan virus (HNTV), Seoul virus (SEOV), YFV, and KFDV. Oligonucleotide primers were designed in conserved genomic regions to detect the broadest number of members for a given pathogen species. We developed a software program that culls sequence information from GenBank, performs multiple alignments with ClustalW, and designs primers optimized for multiplex PCR. The program uses a greedy algorithm to identify conserved sequences and create the minimum set of primers for amplification of all sequences in the alignment. Primers are selected within standard design constraints whenever possible (melting temperature 55°C–65°C, guanine-cytosine content 40%–60%, no hairpins); degenerate positions are introduced in cases where template divergence requires more flexibility. Although degeneracy is not tolerated in the five 3´ nucleotides, MassTag PCR allows up to 4 nonneighboring variable positions per primer. Primers are checked by the basic local alignment search tool for potential hybridization to sequenced vertebrate genomes (Table 1).

**Table 1:** Greene MassTag panel VHF version 1.0*

Target	MassTag	Name	Sequence	Gene
ZEBOV	718 (fwd)	EboZA-U234	AACACCGGGTCTTAATTCTTATATCAA	L
	646 (rev)	EboZA-L319	GGTGGTAAAATTCCCATAGTAGTTCTTT	
SEBOV	503 (fwd)	EboSU-U416	CGAGCCTAACGTTTTGGGC	L
	630 (rev)	EboSU-L489	GCTCCAGGAATTGTTCGGGTA	
MARV	654 (fwd)	MARV-U12816C	CCCTCCATATCTTAGACAACATATTGTG	L
	395 (rev)	MARV-L12994	CCCAACACTCCTGGTTCACAGC	
LASV†	558 (fwd)	Las4-U92	ACTGCATTYTCATACTTYCTRGAATC	NP
	686 (rev)	Las4-L257	CCRGGYTTGACCAGTGCTGT	
RVFV	658 (fwd)	RVF-U578	GGATTGACCTGTGCCTGTTGC	N
	495 (rev)	RVF-L660	GCATTAGAAATGTCCTCTTTTGCTGC	
CCHFV	499 (fwd)	CCHV-U4	AGAAACACGTGCCGCTTACGCCCA	N
	710 (rev)	CCHV-L120	CCATTTCCYTTYTTRAACTCYTCAAACCA	
HNTV	479 (fwd)	HAN-U179	AYACAGCAGCAGTTAGCCTCCT	N
	702 (rev)	HAN-L245	GCT GCC GTA RGT AGT CCC TGTT	
SEOV	455 (fwd)	SEO-U243	CAGGATTGCAGCAGGGAAGA	N
	602 (rev)	SEOUL-L309	ATGATCACCAGGYTCTACCCC	
YFV	467 (fwd)	YF-U186	GCTGGGAGCGCGGTATC	NS5
	670 (rev)	YF-L249	GGAAGCCCAATGGTCCTCAT	
KFDV	483 (fwd)	KYF-U170	TGGAAGCCTGGCTGAAAGAG	NS5
	614 (rev)	KYF-L233	TCATCCCCACTGACCAGCAT	

*ZEBOV, Ebola Zaire virus; SEBOV, Ebola Sudan virus; MARV, Marburg virus; LASV, Lassa virus; RVFV, Rift Valley fever virus; CCHV, Crimean-Congo hemorrhagic fever virus; HNTV, Hantaan virus; SEOV, Seoul virus; YFV, yellow fever virus; KFDV, Kyasanur Forest disease virus; fwd, forward; rev, reverse. †Primers were designed on Lassa lineage IV sequences (*15*) and the recently identified outlier sequence Lassa AV (AF256121).

Because only released mass tags are analyzed, staggering the size of amplification products created in multiplex reactions is unnecessary; thus, primers are selected for efficient and consistent performance irrespective of amplicon size (typically 80–200 bp). Before committing to synthesis of tagged primers, the functionality of candidate multiplex primer panels is examined in a series of amplification reactions that use prototype templates representing individual microbial targets. Primers that fail to yield a single, specific product band in agarose gel analysis are replaced. Target sequence standards for evaluation are cloned into pCR2.1-TOPO (Invitrogen, Carlsbad, CA, USA) by using PCR amplification of cDNA templates obtained by reverse transcription (RT) of extracts from infected, cultured cells or by assembly of overlapping synthetic polynucleotides.

The agents assayed in the VHF panel have RNA genomes; thus, assay sensitivity was determined by using synthetic RNA standards. Synthetic RNA standards were generated from linearized target sequence plasmids by using T7 polymerase (mMessage mMachine, Invitrogen). After quantitation by UV spectrometry, RNA was serially diluted in 2.5 μg/mL yeast tRNA (Sigma, St. Louis, MO, USA), reverse transcribed with random hexamers by using Superscript II (Invitrogen), and analyzed by MassTag PCR as previously described (*14*). QIAquick 96 PCR purification cartridges (Qiagen, Hilden, Germany, with modified binding and wash buffers) were used to remove unincorporated primers before tags were decoupled from amplification products by UV photolysis in a flow cell and analyzed in a quadrapole mass spectrometer by using positive-mode atmospheric pressure chemical ionization (APCI-MS, Agilent Technologies, Palo Alto, CA, USA). The sensitivity of the 10-plex VHF panel with synthetic RNA standards was <50 RNA copies per assay (Table 2). Sensitivity and specificity of multiplex primer panels is assessed empirically by using calibrated synthetic standards as well as tissue culture–derived viral nucleic acid for each assembled panel.

**Table 2:** Sensitivity of detection with synthetic RNA standards

Pathogen*	Detection threshold (RNA copies)†
ZEBOV	20
SEBOV	20
MARV	20
LASV	20
RVFV	20
CCHFV	50
HNTV	20
SEOV	50
YFV	20
KFDV	20

*ZEBOV, Ebola Zaire virus; SEBOV, Ebola Sudan virus; MARV, Marburg virus; LASV, Lassa virus; RVFV, Rift Valley fever virus; CCHV, Crimean-Congo hemorrhagic fever virus; HNTV, Hantaan virus; SEOV, Seoul virus; YFV, yellow fever virus; KFDV, Kyasanur Forest disease virus. †RNA copies refers to the number of molecules subjected to reverse transcription; half of the reverse transcription reaction was then used for polymerase chain reaction amplification.

Tissue culture extracts were used to examine assay specificity. Random primed cDNA obtained from cultures of ZEBOV, SEBOV, MARV, YFV isolates from the Gambia and Côte d’Ivoire, RVFV, CCHFV, HTNV, SEOV, and LASV strains Josiah, NL, and AV were subjected to mass tag analysis. In all instances, only the appropriate cognate mass tags were detected (data not shown). No spurious signal was identified in assays with water or RNA controls.

Performance with clinical materials was tested by using blood, sera, or oral swabs from 24 human patients of VHF previously diagnosed through virus isolation, RT-PCR, or antigen detection enzyme-linked immunosorbent assay. Differential diagnosis by blinded MassTag PCR analysis was accurate in all cases (Table 3). For the samples from the 2005 Angola Marburg outbreak the result of MassTag PCR was similar to that of diagnostic single-plex PCR. ZEBOV sample 5004, obtained on day 17 of illness when serologic test results were positive for immunoglobulin M (IgM) and IgG, was negative by viral culture but positive in MassTag PCR.

**Table 3:** MassTag polymerase chain reaction analysis of clinical specimens from viral hemorrhagic fever patients*

Previous diagnosis	Sample identification	Sample type	Year/origin	MassTag result†
ZEBOV	5015	Serum	1995/Kikwit, DRC	+++, ZEBOV
ZEBOV	5014	Serum	1995/Kikwit, DRC	+++, ZEBOV
ZEBOV	5004	Serum	1995/Kikwit, DRC	+++, ZEBOV
ZEBOV	6317	Serum	1995/Kikwit, DRC	+++, ZEBOV
ZEBOV	6313	Serum	1995/Kikwit, DRC	+++, ZEBOV
MARV	246-00-5	Hemolyzed whole blood	2000/Durba, DRC	+, MARV
MARV	226-00-4	Hemolyzed whole blood	2000/Durba, DRC	++, MARV
MARV	246-00-7	Hemolyzed whole blood	2000/Durba, DRC	+, MARV
MARV	98-00-2	Hemolyzed whole blood	2000/Durba, DRC	+++, MARV
MARV	461	Blood	2005/Uige, Angola	+++, MARV
MARV	462	Oral swab	2005/Uige, Angola	+++, MARV
MARV	475	Blood	2005/Uige, Angola	++, MARV
MARV	476	Oral swab	2005/Uige, Angola	+, MARV
LASV	98-04-1	Serum	2004/Sierra Leone	+++, LASV
LASV	98-04	Serum	2004/Sierra Leone	++, LASV
LASV	98-04-5	Serum	2004/Sierra Leone	+, LASV
LASV	80-04-1	Serum	2004/Sierra Leone	+++, LASV
RVFV	98002009	Serum	1998/Kenya	+, RVFV
RVFV	H6061989	Serum	1998/Kenya	+, RVFV
RVFV	98002019	Serum	1998/Kenya	++, RVFV
RVFV	77-04	Serum	2004/Namibia	++, RVFV
CCHFV	187-86	Serum	1986/South Africa	+, CCHFV
CCHFV	30-93	Serum	1993/South Africa	+++, CCHFV
CCHFV	465-88	Serum	1988/South Africa	+++, CCHFV
CCHFV	407-89	Serum	1989/South Africa	+++, CCHFV
CCHFV	215-90	Serum	1990/South Africa	++, CCHFV

*ZEBOV, Ebola Zaire virus; MARV, Marburg virus; LASV, Lassa virus; RVFV, Rift Valley fever virus; CCHV, Crimean-Congo hemorrhagic fever virus; DRC, Democratic Republic of Congo. †Relative ranking of results: +, signal-to-noise ratio <4; ++, signal-to-noise ratio >4 and <8; +++, signal-to-noise ratio >8.

## Conclusions

These results confirm earlier work in respiratory diseases that show that MassTag PCR offers a rapid, sensitive, specific, and economic approach to differential diagnosis of infectious diseases. Small, low-cost, or mobile APCI-MS units extend the applicability of this technique beyond selected reference laboratories. Given the capacity of the method to code for up to 32 genetic targets, we are expanding the hemorrhagic fever panel to include additional viruses (dengue and South American hemorrhagic fever viruses) and are exploring the inclusion of bacterial and parasitic agents that may result in similar clinical signs and symptoms and, thus, have to be considered in differential diagnosis.
